# Comparative Analysis of Different Serological and Molecular Tests for the Detection of Small Ruminant Lentiviruses (SRLVs) in Belgian Sheep and Goats

**DOI:** 10.3390/v10120696

**Published:** 2018-12-08

**Authors:** Rodolphe Michiels, Eva Van Mael, Christian Quinet, Nadjah Radia Adjadj, Ann Brigitte Cay, Nick De Regge

**Affiliations:** 1 Unit of Enzootic, Vector-Borne and Bee Diseases, Sciensano, Groeselenberg 99, 1180 Brussels, Belgium; nadjahradia.adjadj@sciensano.be (N.R.A.); annbrigitte.cay@sciensano.be (A.B.C.); nick.deregge@sciensano.be (N.D.R.); 2 Dierengezondheidszorg Vlaanderen (DGZ), Industrielaan 29, 8820 Torhout, Belgium; eva.vanMael@dgz.be; 3 Association Régionale de Santé et d’Identification Animales (ARSIA), Allée des Artisans 2, 5590 Ciney, Belgium; christian.quinet@arsia.be

**Keywords:** Maedi-Visna virus, caprine arthritis encephalitis virus, small ruminant lentiviruses, diagnosis, ELISA, agar gel immunodiffusion, qPCR, Belgium, control programme

## Abstract

Countries rely on good diagnostic tests and appropriate testing schemes to fight against economically important small ruminant lentivirus (SRLV) infections. We undertook an extensive comparative analysis of seven commercially available serological tests and one in-house real-time PCR (qPCR) detecting genotype A and B strains using a large panel of representative Belgian field samples and samples from experimentally infected sheep and goats. ELISAs generally performed well and detected seroconversion within three weeks post experimental infection. Two enzyme-linked immunosorbent assays (ELISAs) (Elitest and IDscreen^®^ kits) showed the highest sensitivities (>96%) and specificities (>95%) in both species, and their combined use allowed to correctly identify the infection status of all animals. Individual agar gel immunodiffusion (AGIDs) kits lacked sensitivity, but interestingly, the combined use of both kits had a sensitivity and specificity of 100%. qPCRs detected SRLV infection before seroconversion at two weeks post infection and showed a specificity of 100%. Sensitivity however remained suboptimal at 85%. These results allow to propose a faster and cheaper diagnostic testing strategy for Belgium by combining a first ELISA screening, followed by confirmation of positive samples in AGID and/or a second ELISA. Since genotypes A and B strains are predominant in many countries, these results are interesting for other countries implementing SRLV control programs.

## 1. Introduction

Small ruminant lentiviruses (SRLV) are a group of retroviruses causing a persistent multisystemic and fatal disease in sheep and goats. This viral continuum includes the formerly known Maedi-Visna virus (MVV) described in sheep and the caprine arthritis encephalitis virus (CAEV) described in goats [1]. SRLVs are nowadays classified into five genotypes, from A to E [2,3,4]. Genotype A and B strains, which are sometimes referred to as MVV-like and CAEV-like, respectively, are widely distributed, while genotype C, D, and E strains were only found in more restricted areas [5,6,7]. Previous phylogenetic studies have demonstrated that these viruses are capable to cross the species barrier between sheep and goats [2,8,9,10].

SRLV infections are mostly transmitted at an early age when kids suckle milk and colostrum from their infected mother. Less efficient SRLVs can also disseminate by air when animals are housed in close contact in intensive production systems [11,12,13]. Once SRLVs are transmitted and they cross the mucosal surface, they target monocytes and macrophages where they integrate as provirus in the host cell genome. Through the circulation, stem cells or precursors cells from the bone marrow get infected by circulating macrophages allowing the establishment of a life-long infection in the animal [1].

After an incubation period of several years, up to 30% of the SRLV infected animals will start to suffer from degenerative lesions in joints, mammary glands, and lungs [14,15,16]. Besides the impact on animal welfare, SRLV cause considerable economic losses in the small ruminant industry due to early culling, reduced milk production, and animal movement restrictions [3].

No vaccines or therapies are currently available and the control of SRLV mostly relies on strategies to prevent the introduction of the virus in the herd or transmission from infected mothers to her newborns. These preventive actions rely on good diagnostic methods that ideally should have perfect sensitivity, specificity, and allow for an early detection of SRLV positive animals [17]. In routine diagnostics, the most frequently used assays are the agar gel immunodiffusion (AGID) and the enzyme-linked immunosorbent assays (ELISA). AGID is considered to be highly specific and reproducible, but it has a relative low sensitivity [18,19]. ELISA, on the other hand, is known to be cheaper, can be automated, works for a variety of diagnostic matrices (milk, serum, semen), and allows quantitative interpretations. These advantages make that ELISAs are used as the method of choice for monitoring and large surveillance programs [17,20,21]. Despite the good performance of ELISA tests, none of them allows for detecting all infected animals. Many indirect and competitive ELISAs have been developed using antigens of one or multiple strains, but new strains continue to emerge and thereby reduce the detection range of individual kits. In addition, the slow seroconversion of newly infected animals [22], the tendency for fluctuating antibody response during the first month post-infection [23,24] and individual differences between sheep and goats in the development of the immune response against SRLV antigens [23] make that negative results in ELISA, or in other serological tests, sometimes do not reflect the correct infection status of an animal [21,25]. Besides issues with imperfect sensitivity, also false positive results in ELISA cause major problems in control programs, leading to the incorrect suspension of the SRLV free status and necessitating time and money to perform confirmatory testing.

In addition to serological tests, real-time PCR (qPCR) assays have been developed to detect the presence of viral nucleic acid in tissue and blood. qPCR can detect SRLV infection prior to the antibody response and in theory allows for detecting the latent provirus during the entire lifespan of the animal [21]. Although considered to be highly specific, the sensitivity of this technique can be hampered by the high genetic heterogeneity between strains and the low proviral load existing in infected animals [26]. To improve sensitivity, new qPCRs have been developed over the years using new primers and probes to include more strains from various geographical areas [20,21,27].

In Belgium, a voluntary control program has been implemented that offers the possibility to obtain a SRLV-free certification. To receive the SRLV free status, farmers regularly have to show the negative status of their flocks. The current testing protocol is based on an initial screening where each animal is tested in one ELISA (Elitest, Hyphen Biomed, Neuville-sur-Oise, France) that was shown to have good test characteristics in previous studies. ELISA positive samples are then tested by a combination of two AGIDs (Maeditect kit, Apha Scientific, Addlestone, Surrey, United Kingdom; AGID CAEV P28, Idexx, Westbrook, ME, USA) for confirmation [28]. When the ELISA result is not confirmed by the AGID, a new serum and whole blood sample need to be collected from the suspected animal, which are then tested in ELISA, AGID, and qPCR before a final decision on the infection status of the animal is taken [4]. In 2014, 508 sheep farmers and 27 goat farmers participated to the voluntary program, and although it functions well, regular problems are experienced [4]. These mostly relate to some unexpected positive ELISA results in flocks that are certified SRLV-free for multiple years and are suspected to be false positive reactions. These positive results, together with the temporal suspension of the certificate and the time and costs associated with the collection of extra samples for confirmatory testing, causes frustration among participants, making it that farmers regularly drop out of the program.

To improve the testing protocol of our national control program, we decided to implement an extensive comparative analysis of commercially available tests for SRLV detection in sheep and goat. Our analysis includes recently developed tests that were not thoroughly investigated before and uses a large number of samples from different backgrounds. Besides serological tests, such as AGID and ELISAs, also two qPCRs were included and the possibility to use blood clots as an alternative to PBMCs for molecular SRLV detection was evaluated. Good results with the latter matrix would allow for performing both serological and molecular SRLV detection on one single blood sample, thereby reducing the time and costs to obtain final results. The present study will not only be helpful for the Belgian national authorities in the fight against SRLV, but also for other countries that are willing to implement or improve their control programs for SRLV infections.

## 2. Materials and Methods

### 2.1. Diagnostic Samples

In total, three panels of sheep and goat samples were collected and tested. An overview of the sample cohorts can be seen in Supplementary Table S2. They included:

Panel 1: Leucocytes and serum samples from 553 sheep from 87 farms and 394 goats from 76 farms sampled in the context of a nationwide SRLV seroprevalence study in Belgium carried out between November 2015 and May 2016. A stratified random sampling of farms was applied proportional to the number of sheep and goat farms per province. Per farm, a maximum of seven animals of at least one year old were randomly selected. Details on the sampling strategy and sample collection can be found in Michiels et al. [4]. After qPCR testing of all leucocyte pellets, a subsample of 125 blood clots was selected and tested in qPCR. This selection included blood clots from all 51 and 24 seropositive sheep and goats, respectively, found in panel 1, plus randomly selected blood clots from both 25 seronegative sheep and goats of panel 1 that were found seronegative.

Panel 2: 50 SRLV positive serum samples from 29 goats and 21 sheep picked from our routine diagnostic (i.e., other than panel 1). These sera that originated from different farms were previously found positive in the AGID-CAEV p28 kit (Idexx).

Panel 3: Sequential sera and leucocytes that were collected during an experimental infection of two sheep and one goat aged between one and two year old. These SRLV negative animals were intratracheally inoculated with an SRLV strain that was isolated from a naturally infected sheep positive in the genotype A qPCR and an SRLV strain isolated from a naturally infected goat positive in the genotype B qPCR (see further). Both virus stocks were produced via the co-cultivation of peripheral blood mononuclear cells (PBMCs) from these naturally infected animals (see 2.2.3) with primary sheep choroid plexus cells, using methods that were described by Petursson and colleagues, 1976 [29]. Sequencing and phylogenetic analysis using the methods described by Shah et al., 2004 [2] confirmed that the isolates belonged to genotype A and B, respectively.

After local anesthesia of the tracheal zone with 2 mL of Lidocaïne 4% + adrenaline, the SRLV negative sheep and goat were intratracheally inoculated with 1ml of the genotype A (10^2.5^ TCID_50_/mL) or B (10^3.66^ TCID_50_/mL) virus stocks, respectively. To ensure the success of the inoculation, animals were observed for coughing immediately after inoculation and the rectal temperature was monitored for 10 days to check for any signs of infection. The inoculated sheep and goat were bled every week until eight and seven weeks post infection, respectively, to produce serum and leucocyte pellets and they were euthanized at the end of this study period. The study protocol and experimental procedure were approved by the national Ethical Commission of the CODA-CERVA/WIV-ISP under the approval 20170529-01.

### 2.2. Sample Preparation

#### 2.2.1. Serum—Blood Clots

Blood was collected in 10 mL serum tubes in order to obtain serum and blood clots. Tubes were spun at 1500 rpm for 10 min and sera and blood clots were frozen separately at −20 °C until testing. For DNA extraction from blood clots, about 0.5 cm^3^ of the blood clots was cut and transferred into a 2 mL eppendorf containing zirconia beads and one steel bead. 1 mL of ATL buffer (Qiagen, Hilden, Germany) was added and the blood clots were homogenized in a TissueLyser II (Qiagen, Hilden, Germany) for 5 min at 25 Hz. After homogenization, tubes were centrifuged at 10,000 rpm for 1 min and the supernatant was used for DNA extraction.

#### 2.2.2. Leucocyte Pellets

Leucocytes pellets were prepared from whole blood samples. 1.5 mL of blood was added to 8.5 mL of hemolysis buffer (16.6 g NH_4_Cl, 2.0 g NaHCO_3_, 0.185 g EDTA disodium salt per L H_2_O; pH 7.4) [30]. After 20 min of incubation at room temperature, samples were centrifuged for 10 min at 3000× *g* and the pellet was resuspended in 200 µL of phosphate buffer saline (PBS).

#### 2.2.3. PBMCs

Peripheral blood mononuclear cells (PBMCs) from a naturally genotype A infected sheep and from a naturally genotype B infected goat were used to obtain SRLV isolates for the experimental infection study (see 2.1). PBMCs were isolated from 15 mL of heparinized blood diluted in 15 mL of RPMI-1640 (Thermo Fisher Scientific, Waltham, MA, USA) and were slowly overlaid on 15 mL of Histopaque—1077 in a 50 mL tube (Sigma-Aldrich, St. Louis, MO, USA). Tubes were centrifuged at 2000 rpm for 25 min at room temperature. After separation, PBMCs were aspirated from the buffy coat layer and diluted in 30 mL of RPMI-1640. Cells were washed twice with RPMI-1640 and plated at a concentration of 10^6^ cells/mL in a six-well plate. PBMCs were cultivated at 37 °C for 10 days and the culture medium consisted of RPMI-1640 supplemented with 1 mM glutamine, 10 mM HEPES, 0.1 mM 2-mercapto ethanol, 1% gentamicin, 2% fungizone, 1000 IU/mL penicillin, and 20% fetal calf serum. This specific medium that was adapted from Gorrell et al. was necessary to stimulate the differentiation of monocytes into macrophages and activate the viral replication [31].

### 2.3. Serological Tests

Serum samples from panels 1, 2, and 3 were tested for the presence of SRLV antibodies in five commercial ELISA kits (Elitest MVV/CAEV (Hyphen Biomed, Neuville-sur-Oise, France), MVV/CAEV p28 Ab screening test (Idexx, Westbrook, ME, USA), ID screen MVV/CAEV indirect (IDvet, Grabels, France), LSIVet ruminant Maedi-Visna/CAEV serum ELISA kit (LSI, Thermo Fisher Scientific, Waltham, MA, USA), and Eradikit SRLV screening test (IN3 diagnostic, Torino, Italia)) and in two commercial AGID tests (AGID-CAEV p28 kit (Idexx, Westbrook, ME, USA) and Maeditect kit (Apha Scientific, Addlestone, Surrey, United Kingdom)). Supplementary Table S1 provides the available details on the antigens used in the different tests. Samples from panel 1 were tested in ELISA by the two regional Belgian animal health laboratories: “Dierengezondheidszorg Vlaanderen” and “Association Régionale de Santé et d’Identification Animales”. The ELISAs for panels 2 and 3 and the AGID tests on samples from panels 1, 2, and 3 were carried out in the national reference laboratory for SRLV, which is part of the Belgian public and animal health center “Sciensano”. For ELISAs, samples were considered as positive when the optical density (OD) or S/P values were equal or above the cut-off value that was calculated from the manufacturer’s instructions. Tests were only validated if the controls samples included in the kit fulfilled the prescribed conditions.

### 2.4. DNA Extraction and Molecular Tests

qPCR was used to detect the presence of SRLV nucleic acids in leucocyte pellets from samples of panel 1 and 3 and in a selection of blood clots from panel 1 (see 2.1). DNA extraction from the leucocyte pellets and the supernatants of homogenized blood clots was performed using the QIAmp DNA minikit (Qiagen, Hilden, Germany), following the manufacturer’s instructions and DNA was eluted in 100 µL of elution buffer. All DNA extracts were tested in two “in-house” qPCRs targeting the *gag* gene region of SRLV that were designed to detect genotype A and B strains. Primers and probes for the detection of genotype A strains were previously described [27]. For genotype B strains, following primers and probe were designed based on an in silico study: forward (5′-AAWCCGCCRTGGTGARTCTAGATA-3′); reverse (5′-CSAGYTCAGGATAATCYCKTTTCC-3′); SRLV-B probe (5′ 56-FAM-TGGCGAGGCARGTCTCCGG-3IABkFQ). Each sample was also tested for the presence of *β-actin* to control the DNA extraction. The FastStart TaqMan Probe Master kit (Roche, Basel, Switzerland) was used for qPCR reactions. For each reaction, a master mix consisting of 4 μL RNase free water, 10 μL 2× FastStart probe master buffer and 1 μL of a pre-mix containing the forward primer (10 μM), reverse primer (10 μM), and probe (4 μM) was prepared. 5 μL of DNA extract was added per reaction. The following amplification program was used: 10 min at 95 °C, followed by 45 cycles of 15 s at 95 °C and 45 s at 60 °C. All qPCRs were carried out on a LightCycler 480 Real-Time PCR system (Roche, Basel, Switzerland) at the national public health center “Sciensano”. Negative extractions controls and negative and positive amplification controls (DNA extracts from lung of SRLV positive and negative sheep and goats) were included in each run. Samples that were associated with a characteristic amplification curve that sorted a Ct value between 40 and 45 were considered to be positive and are mentioned as Ct > 40.

## 3. Results

### 3.1. Allocation of an Infection Status to Each Animal

To accommodate for the absence of a gold standard test for SRLV detection, we decided to define an infection status for each animal of sample panel 1 by considering all obtained serological results. A similar approach has already been applied in previous studies [28]. Besides the five ELISA results, we also considered one combined result of both AGID tests, whereby a sample was considered positive in AGID if it gave a positive reaction in at least one of both AGID tests. 84.5% of samples showed to have identical results in all serological tests, 14.7% had only one deviating result, while less than 1% of the samples had two deviating results (Table 1). Each animal received the status that was found in the majority of tests (result found in ≥4 tests). The fact that high agreement amongst tests was found for almost all samples indicates that we can have confidence that the final infection status allocated to each animal reflects its true infection status. In total, 75 animals (51 sheep and 24 goats) were qualified as positive and 872 (502 sheep and 370 goats) were qualified as negative for SRLV infection.

### 3.2. Sensitivity and Specificity of Serological Tests

Sample panel 1 was used to determine the sensitivity and specificity of all tests by comparing the individual test results to the final infection status of each animal determined, as described above. A detailed summary of these results is shown in Table 2.

For AGID, low sensitivities of 35% in sheep and 75% in goats were observed when using the AGID CAEV P28 kit alone. The Maeditect kit showed higher sensitivities of 100% in sheep and 87.5% in goats. Interestingly, when the results of both AGID kits were combined, and a positive result obtained in one of both kits was considered to be sufficient to have a positive AGID result, a sensitivity of 100% was observed in sheep and goats (Table 2). The specificity of both AGID kits was 100%.

For ELISA, the highest sensitivities in sheep sera were observed with the Elitest MVV/CAEV (98.0%) and the ID screen^®^ MVV/CAEV indirect (100%) kits. Considerable lower sensitivities were found with the LSIVet™ Ruminant Maedi-Visna/CAEV serum ELISA kit (90.2%) and the MVV/CAEV p28 Ab screening test kit (84.3%). In goat samples, a sensitivity of 100% was found with three ELISA kits, including the ID screen^®^ MVV/CAEV indirect, LSIVet™ Ruminant Maedi-Visna/CAEV serum ELISA kit, and the Eradikit™ SRLV screening test (Table 2). Also here, the lowest sensitivity was observed with the MVV/CAEV p28 Ab screening test kit (91.7%). Where the MVV/CAEV p28 Ab screening test kit was the least sensitive test in both species, it showed to have the highest specificities in sheep (99.6%) and goats (100%). Only the LSIVet™ Ruminant Maedi-Visna/CAEV serum ELISA kit had a markedly reduced specificity of 92.8% in sheep with 36 false positive samples and 85.7% in goat with 53 false positive samples (Table 2).

Since only 75 positive animals were present in sample panel 1, sample panel 2 containing 50 samples found positive in AGID during our routine diagnostics was tested in the five ELISAs to further evaluate their sensitivity. The results for sera of panel 2 were in line with the results of sample panel 1. The ID screen^®^ MVV/CAEV indirect kit showed the highest sensitivity (100%), followed by the Elitest MVV/CAEV (98%) and the Eradikit™ SRLV screening test kits (94%). The lowest sensitivity was again observed with the MVV/CAEV p28 Ab screening test kit (88%) (Table 3).

### 3.3. SRLV-Specific Antibody Detection in Sequential Sera from Experimentally Infected Small Ruminants

To evaluate differences in the capacity of the different serological tests to detect SRLV-specific antibodies in sera collected at well-defined time points early post SRLV infection, we used samples originating from an experimental infection of two sheep and one goat inoculated with a genotype A strain and a genotype B strain, respectively.

All kits detected SRLV-specific antibodies early post experimental infection (Table 4). Some differences in the time that is needed to detect seroconversion after experimental infection between the different kits were found, but the low number of animals and the known individual variation in the development of immune response between animals [16,23] make that no general conclusions on this aspect can be drawn. In the experimentally infected goats, the Eradikit™ SRLV screening test kit was the only test to detect the presence of SRLV specific antibodies already at 2 weeks post infection, while all other kits detected seroconversion at three weeks pi. In the experimentally infected sheep, the AGID CAEV P28 kit and three ELISAs kits, including the Elitest MVV/CAEV, MVV/CAEV p28 Ab screening test, and LSIVet™ Ruminant Maedi-Visna/CAEV serum ELISA kit showed a comparable performance and detected antibodies at 21 days pi. While the Eradikit™ SRLV screening test kit was the most sensitive test to detect seroconversion in goat, it only detected seroconversion at 35 and 49 days pi in both sheep, respectively.

Interestingly, although both sheep were infected with the same inoculum, a different evolution of the humoral immune response seems to have occurred in both animals. Sheep 1 first reacted with the AGID test using the -CAEV p28 capsid protein as antigen, while sheep 2 was first found positive in the AGID kit using the MVV gp135 transmembrane protein as antigen. Also differences of up to three weeks to detect seroconversion in both animals were found in some ELISA kits (e.g., LSIVet™ Ruminant Maedi-Visna/CAEV serum ELISA kit).

### 3.4. Molecular SRLV Detection in Leucocyte Pellets

Leucocyte pellets of 536 sheep and 387 goats from sample panel 1 were tested in qPCR for the presence of genotypes A or B strains. Seventeen sheep and seven goats samples were excluded from the testing due to their poor blood quality based on β-actin test results. qPCRs showed to be highly specific in both sheep (100%) and goats (100%) samples. Sensitivity was however suboptimal with 88.0% in sheep and 83.3% in goats (Table 5). In total, 44 sheep reacted positively in the genotype A qPCR. For goats, there were six positives in genotype A qPCR and 18 in the genotype B qPCR. Interestingly, four goats reacted positive in both qPCRs, indicative of a co-infection.

When looking at the results from the experimental infection (Table 4), two out of three animals were qPCR positive at two weeks p.i., the other at four weeks p.i. In general, the Ct values were high (>35), indicating low viral loads (Table 6). Ct values >40 were considered positive, since they were accompanied with a characteristic amplification curve. No amplification curve was ever observed in the leucocyte pellets of seronegative animals from panel 1. In experimentally infected sheep 1, SRLV proviral DNA was only intermittently detected.

### 3.5. Molecular SRLV Detection in Blood Clots

To analyse whether blood clots could be used as a matrix for molecular SRLV detection, 125 blood clots and corresponding leucocyte pellets (76 from sheep and 49 from goats) were tested in qPCR. All blood clots from seronegative animals (25 from sheep and 25 from goats) tested negative in both matrices. While it was shown in point 3.4 that the qPCR on leucocyte pellets already had a suboptimal sensitivity as compared to serology, this was even worse for qPCR on blood clots. We found a relative sensitivity of SRLV detection by qPCR in blood clots of only 63% and 21% compared to serology in sheep and goats, respectively (Table 7. Similar low relative sensitivities of 75% and 25% were found if qPCR results on blood clots were compared to qPCR results on leucocyte pellets in sheep and goats, respectively (Table 7). Its currently unclear why this relative sensitivity is significantly lower (Fisher exact test; *P* = 0.008) in goat samples as compared to sheep samples. The average Ct value in blood clots was four Cts higher than in leucocytes pellets for animals that were positive in both matrices.

## 4. Discussion

Due to economic consequences of SRLV infections and the absence of vaccines, multiple countries, including Belgium, have implemented voluntary or obligatory control programs [4,17,25,32,33,34,35]. These control programs are essential to detect and eliminate SRLV infected animals from flocks and their success mostly relies on good diagnostic tools [17]. The applied testing protocols differ between countries and use (combinations of) commercial or in-house produced tests. In Belgium, an indirect ELISA (Elitest MVV/CAEV, Hyphen Biomed, Neuville-sur-Oise, France) is used as a first screening tool, and positive ELISA results need to be confirmed in AGID. If the positive ELISA result is not confirmed in AGID, a new serum and whole blood sample is collected from the suspected animal and tested in ELISA, AGID, and qPCR before a final infection status is accorded to the suspected animal [4]. This protocol has shown good performance but reoccurrence of SRLV infection in certified flocks indicates that a small percentage of infected animals remain undetected. On the other hand, occasional false positive results during the first line ELISA screening cause time consuming confirmatory analyses and frustration among participants. Therefore, we performed an extensive comparison of diagnostic tests for SRLV detection in the Belgian sheep and goat population in order to potentially identify new diagnostic strategies for SRLV detection. We evaluated five ELISAs, two AGIDs, and one in-house qPCRs detecting genotype A and B strains.

In line with previous results, individual AGID tests showed to be highly specific but they had a reduced sensitivity [20,25]. Especially, the AGID based on CAEV p28 protein performed rather poorly. Besides fluctuation in antibody response over time [23,24] and differences in the development of humoral response between individual animals [23], the most probable explanation for this observation is the fact that antibodies against the capsid arise early in the infection and tend to decline later in the infection [36,37,38] On the contrary, antibodies against the envelope glycoproteins, such as gp135, which are used in the Maeditect kit, are present at a later stage of the infection [25,36]. Since the samples from the naturally infected animals (panel 1) were collected from animals of at least one year old, the positive field samples most probably did not originate from recently infected animals, making that it was to be expected that more animals would react positive with the Maeditect kit than with the AGID CAEV P28 kit. Furthermore, it is interesting to observe that more goat samples reacted positive with the AGID kit based on MVV antigen than with the kit using the CAEV antigen. This confirms the serological cross-reactivity induced by SRLV infection in sheep and goats [19], and potentially indicates that some CAEV strains present in Belgium originate and have evolved from an ancient Maedi-Visna virus that was successfully transmitted from a sheep to a goat [39,40]. An important outcome of this study was the finding that combining the results of both AGID tests resulted in 100% sensitivity and specificity of SRLV detection in both sheep and goats. Although AGID tests remain too labor intensive to serve as first line screening tests, the combination of both AGIDs seems to constitute an appropriate confirmatory test. It will be interesting to analyze in the future whether the antigens of both tests could be combined to produce one AGID test with perfect test characteristics.

When looking at the ELISAs, an overall good performance was observed. The highest sensitivities (>96%) in both sheep and goat samples were found with kits that use multiple antigens (Elitest MVV/CAEV, ID screen^®^ MVV/CAEV indirect, Eradikit™ SRLV screening test). These kits also showed good specificity (>95%). Boshoff et al. reported before that the combined use of envelop and capsid antigens resulted in the best ELISA results [41] and the Elitest MVV/CAEV kit was also found as one of the best performing test in a previous comparative study [42]. The highest specificity was found with the MVV/CAEV p28 Ab screening test that only uses one capsid antigen, which seems to strongly decrease the sensitivity of the test.

Both AGID and ELISA tests were capable to detect seroconversion early (i.e., three weeks post infection) after experimental infection. This might indicate that viral replication and the induction of the humoral immune response after intratracheal infection differs from what occurs after natural transmission. More recent studies however also found that lentivirus infections were characterized by rapid seroconversion [23,43]. It is important to consider that only three experimentally infected animals were used in this study and that they were furthermore infected with different SRLV strains and different infectious doses, making that no generalized conclusions on viral replication and SRLV induced immunity can be drawn from this experiment.

Based on the good performance of the ELISA tests, they remain the best choice for the first line screening. For the Belgian situation, where sheep and goat samples are screened using the same protocol and using the same tests, an ELISA that is highly sensitive and specific in both species is preferred. Therefore, the use of the currently used Elitest MVV/CAEV kit seems justified and the ID screen^®^ MVV/CAEV indirect test could be a valid alternative for the first line screening. As indicated above, testing the positive samples found in the first line ELISA screening with the combined AGID test would allow to confirm the positive status of suspected animals or identify potential false positive ELISA results. Alternatively, it could be envisioned to implement a second ELISA as a confirmation test, since this would allow for obtaining confirmatory results in a shorter time span. It is normally advised to use a highly specific confirmatory test when accredited flocks are monitored [25]. Based on our results, the MVV/CAEV p28 Ab screening test kit would thus be most suitable for that purpose. However, if we evaluate this protocol (Elitest MVV/CAEV followed by MVV/CAEV p28 Ab screening test for confirmation of positive results) in a theoretical and a posteriori analysis with our field samples, this does not seem a good option, since too many positive samples from the first screening would be classified as (false) negative (six sheep and one goat) due to the low sensitivity of the ELISA MVV/CAEV p28 Ab screening test kit, which would allow the virus to keep on circulating in the flocks. A better option seems the combination of the Elitest MVV/CAEV kit followed by the ID screen^®^ MVV/CAEV indirect kit as confirmatory test. This sequence of tests allowed for correctly identifying the infection status of all animals in an a posteriori analysis.

As PCRs theoretically should be able to detect SRLV infections before seroconversion and during the entire life span of infected animals [20,44,45], we also evaluated the usefulness of this technique. Early detection before seroconversion was confirmed in samples from our experimental infection, but the level of proviral DNA was generally low and only intermittently detected in one out of three inoculated animals. De Andres et al. already described that the low proviral load observed after seroconversion makes qPCR less sensitive [20]. Our qPCR results on field samples indicate that genotype A and B strains circulate in Belgium and that co-infection with both genotypes occurs. Importantly, our qPCR showed to be highly specific in both sheep and goat samples, but had a suboptimal sensitivity. This is probably related to the high genetic heterogeneity between strains circulating in Belgium and/or the low proviral load in the collected samples [21].

A major disadvantage of the current use of the qPCR as confirmatory test is that a second blood sample needs to be collected from animals that are suspected to be SRLV infected in order to prepare leucocyte pellets, making that time and money is lost before a final status can be determined. We therefore examined whether blood clots, which contain monocytes/macrophages and are available in the tube used for serum sampling, could not be an alternative to leucocyte pellets. This however showed not to be a valid option due to a high loss in sensitivity. This is probably due to a combination of the inhibiting effect of hemoglobin that is present in DNA extracts on the Taq polymerase [46] and the further dilution of infected monocyte/macrophages within the blood clot harboring mostly red blood cells as compared to the amount present in leucocyte pellets. Interestingly, we observed that the relative sensitivity of SRLV detection in blood clots compared to leucocyte pellets was significantly lower in goats compared to sheep, but we cannot provide clear explanation for this observation at this moment.

In conclusion, a first screening with the Elitest MVV/CAEV ELISA kit, combined with both AGID tests and/or the ID screen^®^ MVV/CAEV indirect ELISA kit as a confirmation test seems to be a valid testing protocol for SRLV monitoring and certification in Belgium. It would allow for determining the correct infection status of participating animals and final responses would be obtained faster and cheaper when compared to the current protocol that still includes qPCR as a confirmation test. Although some differences have been observed between genotypes and subtypes circulating in different countries, genotype A and B strains have always been predominant [8], making that these results will also be helpful for other countries thinking of implementing SRLV control programs.

Despite the good results obtained with this proposed protocol in our study, it remains to be seen if it will actually allow to completely eradicate the disease if that would be the goal of an implemented control program. Other diagnostic protocols that succeeded in reducing SRLV prevalence have been confronted with problems once the seroprevalence becomes low at the end of an eradication program [35]. At that moment, other aspects like e.g., low level circulation of genotypes that are not efficiently detected by implemented tests [47], the presence of some individual non-responders that remain undetected and continuously reintroduce the disease [33], reintroduction of SRLV via illegal animal movements, via infected wildlife or via infected sheep in the goat population or vice versa if only eradication in one species is envisioned, etc. make that even with a well validated diagnostic protocol, it becomes difficult to identify the last remaining infected animals. This means that authorities have to continue to invest to prevent reintroduction and to identify and prevent the spread of less prevalent genotypes.

## Figures and Tables

**Table 1:** Agreement among serological test results in order to define the small ruminant lentivirus (SRLV) infection status.

		Sheep		Goats		Total	
		N	%	N	%	N	%
Number of kits with identical results ^a^	6	486	87.9%	314	79.7%	800	84.5%
	5	63	11.4%	76	19.3%	139	14.7%
	4	4	0.7%	4	1.0%	8	0.8%

^a^ Results from both agar gel immunodiffusion (AGID) tests were interpreted together to obtain one final AGID result per animal.

**Table 2:** 2 × 2 contingency tables summarizing individual kit serological test results compared to the defined infection status and derived sensitivity, specificity, and negative/positive predictive values for each test.

	Test Results	Infection Status ^a^			Sheep *N* = 553		Infection Status ^a^			Goats *N* = 394	
		Pos	Neg	Sensitivity	Specificity	PPV/NPV ^c^	Pos	Neg	Sensitivity	Specificity	PPV/NPV ^c^
**Agar Gel Immunodiffusion**											
AGID CAEV P28 kit (Idexx)	Pos	18	0	35.3%	100.0%	100.0%/93.8%	18	0	75.0%	100.0%	100.0%/98.4%
	Neg	33	502				6	370			
Maeditect kit (Apha Scientific)	Pos	51	0	100.0%	100.0%	100.0%/100.0%	21	0	87.5%	100.0%	100.0%/99.2%
	Neg	0	502				3	370			
Total AGIDs ^b^	Pos	51	0	100.0%	100.0%	100.0%/100.0%	24	0	100.0%	100.0%	100.0%/100.0%
	Neg	0	502				0	370			
**ELISAs**											
Elitest MVV/CAEV (Hyphen BioMed)	Pos	50	4	98.0%	99.2%	92.6%/99.8%	23	1	95.8%	99.7%	95.8%/99.7%
	Neg	1	498				1	369			
MVV/CAEV p28 Ab screening test (Idexx)	Pos	43	2	84.3%	99.6%	95.,6%/98.4%	22	0	91.7%	100.0%	100.0%/99.5%
	Neg	8	500				2	370			
ID screen^®^ MVV/CAEV indirect (IDVet)	Pos	51	11	100.0%	97.8%	82.3%/100.0%	24	9	100.0%	97.6%	72.7%/100.0%
	Neg	0	491				0	361			
LSIVet™ Ruminant Maedi-Visna/CAEV serum ELISA kit (LSI)	Pos	46	36	90.2%	92.8%	56.1%/98.9%	24	53	100.0%	85.7%	31.2%/100.0%
	Neg	5	466				0	317			
Eradikit™ SRLV screening test (IN3 diagnostic)	Pos	49	3	96.1%	99.4%	94.2%/99.6%	24	20	100.0%	94.6%	54.5%/100.0%
	Neg	2	499				0	350			

^a^ The SRLV infection status of each animal corresponded to the serological results that was obtained in at least four out of six tests. ^b^ Each sample was considered positive in AGID if it gave a positive reaction in at least one of both AGID tests. ^c^ Positive/negative predictive values.

**Table 3:** Additional evaluation of enzyme-linked immunosorbent assays (ELISA) sensitivity.

	50 Known Positive Samples in AGID		
	Pos	Neg	Sensitivity
Elitest MVV/CAEV (Hyphen BioMed)	49	1	98%
MVV/CAEV p28 Ab screening test (Idexx)	44	6	88%
ID screen^®^ MVV/CAEV indirect (IDVet)	50	0	100%
LSIVet™ Ruminant Maedi-Visna/CAEV serum ELISA kit (LSI)	46	4	92%
Eradikit™ SRLV screening test (IN3 diagnostic)	47	3	94%

**Table 4:** Time to first positive detection by serology and real-time PCR (qPCR) after experimental infections.

	Time to First Positive Detection in Serology and qPCR (Days)							
	AGIDs		ELISAs					qPCR
Animals ^a,b^	AGID CAEV P28 kit (Idexx)	Maeditect kit (Apha Scientific)	Elitest MVV/CAEV (HYPHEN BioMed)	MVV/CAEV p28 Ab screening test (Idexx)	ID screen^®^ MVV/CAEV indirect (IDVet)	LSIVet™ Ruminant Maedi-Visna/CAEV serum ELISA kit (LSI)	Eradikit™ SRLV screening test (IN3 diagnostic)	
Sheep 1	21	42	21	21	35	21	49	14
Sheep 2	>56	35	21	35	21	42	35	28
Goat 1	21	21	21	21	21	21	14	14

^a^ Two sheep and one goat were inoculated with a genotype A and a genotype B strain, respectively. ^b^ Animals were sampled every week until 56 dpi.

**Table 5:** 2 × 2 contingency tables summarizing qPCR results in leucocyte pellets compared to the defined infection status based on six serological tests.

		Test Results	Infection Status ^b^		Sensitivity	Specificity	PPV/NPV ^c^
			Pos	Neg			
qPCRs	Sheep *N* = 536 ^a^	Pos	44	0	88.0%	100.0%	100.0%/98.8%
		Neg	6	486			
	Goats *N* = 387 ^a^	Pos	20	0	83.3%	100.0%	100.0%/98.9%
		Neg	4	363			

^a^ Due to the poor quality of few blood samples, 17 sheep and seven goats were not tested in real-time PCR. ^b^ The SRLV infection status of each animal corresponded to the serological results that was obtained in at least four out six tests. ^c^ Positive/negative predictive values.

**Table 6:** Ct values obtained in leucocyte pellets over time after an experimental inoculation of two sheep with a genotype A strain and one goat with a genotype B strain.

Days p.i	Genotype A		Genotype B
	Sheep 1	Sheep 2	Goat 1
0	Undetected	Undetected	Undetected
7	Undetected	Undetected	Undetected
14	>40 ^a^	Undetected	>40
21	Undetected	Undetected	35.89
28	>40	>40	36.48
35	Undetected	>40	37.16
42	Undetected	>40	38.49
49	>40	37.74	37.11
56	Undetected	37.36	

^a^ Indicating an obtained Ct value between cycle 40 and 45 of the amplification reaction. This Ct value was associated with an actual amplification curve.

**Table 7:** 2 × 2 contingency tables comparing qPCR results obtained in blood clots from sheep and goats to the serological and virological infection status.

			Serological Infection Status ^a^				Virological Infection Status ^b^			
			Pos	Neg	Relative Sensitivity	Relative Specificity	Pos	Neg	Relative Sensitivity	Relative Specificity
Sheep (N = 76)	BC ^c^	Pos	32	0	62.7%	100.0%	32	0	72.7%	100.0%
		Neg	19	25			12	32		
Goats (N = 49)	BC ^c^	Pos	5	0	20.8%	100.0%	5	0	25.0%	100.0%
		Neg	19	25			15	29		

^a^ The SRLV infection status of each animal corresponded to the serological results that was obtained in at least four out six tests. ^b^ The virological SRLV infection status of each animals corresponds to the qPCR status obtained in leucocytes pellets. ^c^ Blood clots.

## Supplementary Materials

Supplementary materials can be found at http://www.mdpi.com/1999-4915/10/12/696/s1.
