# Supplementary material for: Comparative Analysis of Different Serological and Molecular Tests for the Detection of Small Ruminant Lentiviruses (SRLVs) in Belgian Sheep and Goats
Source: Viruses. 2018 Dec 8;10(12):696. doi: 10.3390/v10120696 (PMC6316478; doi:10.3390/v10120696)
Supplement: Supplementary file 1 [file viruses-10-00696-s001.pdf]

Supplementary Table S1. Overview of the different serological test kits used and their characteristics.

	ELISA type	Antigen/ target [a]	Serum volume	Duration [b]
**Agar Gel Immunodiffusion**				
AGID CAEV P28 kit (Idexx)		CAEV capsid p28	63µl	3 days
Maeditect kit (Apha Scientific)		MVV gp135	52µl	3 days
**ELISAs**				
Elitest MVV/ CAEV (Hyphen BioMed)	Indirect	MVV capsid rp25 and gp46 TM	2-4µl	150 min
MVV/ CAEV p28 Ab screening test (Idexx)	Indirect	CAEV capsid p28	10µl	110min
ID screen® MVV/CAEV indirect (IDVet)	Indirect	Panel of MVV/ CAEV TM proteins, p25 and gp135	10µl	90 min
LSIVet™ Ruminant Maedi-Visna/CAEV serum ELISA kit (LSI)	Blocking	Panel of MVV/CAEV proteins	50µl	170 min
Eradikit™ SRLV screening test (IN3 diagnostic)	Indirect	Mix of gag and env peptides belonging to the 3 most divergent SRLV viral genotypes (A, B, E)	5µl	140min

[a] Information about antigen/target coated on the microtiter plate were found either on the manufacturer's website or in literature

[b] Time to result. These durations were estimated based on incubation times provided by the kits. Time to prepare samples, washing and reading steps were not included

Abbreviations: r, recombinant; MVV, Maedi Visna Virus; CAEV, caprine arthritis encephalitis virus; TM, transmembrane; gp, glycoprotein

Supplementary Table S2. Overview of the sample cohorts.

	Origin	N° of animals	Type of samples	Description
Panel 1	Field	553 sheep<br>394 goats	Sera<br>Leucocyte pellets	Samples were collected in the context of a nationwide SRLV seroprevalence study where sheep and goat holders were randomly selected all over Belgium. Farmers included in this study were not participating to the national SRLV control program
Panel 2	Routine diagnostic	21 sheep<br>29 goats	Sera	Positive sera were picked from our routine diagnostic where they were previously found positive with the AGID CAEV P28 kit

Panel 3	Experimental infection	2 sheep<br>1 goat	Sera<br>Leucocyte pellets	Sequential samples were collected during an experimental infection of 2 sheep and 1 goat infected with a genotype A and a genotype B strains respectively. Animals were bled every week for 8 weeks in total.
